# Clinical Considerations of Preimplantation Genetic Diagnosis for Monogenic Diseases

**DOI:** 10.1371/journal.pone.0139613

**Published:** 2015-09-30

**Authors:** Xiaokun Hu, Jing Wang, Yubin Li, Yizi Wang, Chenhui Ding, Yanhong Zeng, Yanwen Xu, Canquan Zhou

**Affiliations:** Center for Reproductive Medicine and Department of Gynecology & Obstetrics, the First Affiliated Hospital of Sun Yat-Sen University, Guangzhou, Guangdong, China; Peiking university third hospital, CHINA

## Abstract

**Purpose:**

The aim of this study was to explore factors contribute to the success of PGD cycles for monogenic diseases.

**Methods:**

During a 3-year period (January 2009 to December 2012), 184 consecutive ICSI-PGD cycles for monogenic diseases reaching the ovum pick-up and fresh embryo-transfer stage performed at the Reproductive Medicine Center of The First Affiliated Hospital Of Sun Yat-sen University were evaluated.

**Results:**

ICSI was performed on 2206 metaphase II oocytes, and normal fertilization and cleavage rates were 83.4% (1840/2206) and 96.2% (1770/1840), respectively. In the present study, 60.5% (181/299) of day 3 good-quality embryos developed into good-quality embryos on day 4 after biopsy. Collectively, 42.9% clinical pregnancy rate (79/184) and 28.5% implantation rate (111/389) were presented. In the adjusted linear regression model, the only two significant factors affecting the number of genetically unaffected embryos were the number of biopsied embryos (coefficient: 0.390, 95%CI 0.317–0.463, P = 0.000) and basal FSH level (coefficient: 0.198, 95%CI 0.031–0.365, P = 0.021). In the adjusted binary logistic regression model, the only two significant factors affecting pregnancy outcome were the number of genetically available transferable embryos after PGD (adjusted OR 1.345, 95% CI 1.148–1.575, P = 0.000) and number of oocyte retrieved (adjusted OR 0.934, 95% CI 0.877–0.994, P = 0.031).

**Conclusion:**

There should be at least four biopsied embryos to obtain at least one unaffected embryos in a PGD system for patients with single gene disorder and under the condition of basal FSH level smaller than 8.0mmol/L. Moreover, if only a low number (< 4) of biopsied embryos are available on day 3, the chance of unaffected embryos for transfer was small, with poor outcome.

## Introduction

Preimplantation genetic diagnosis (PGD) is an established reproductive option for couples at risk of transmitting specific inherited disorders to their offspring. The intended goal of PGD is to select and transfer embryos unaffected after genetic analysis before a clinical pregnancy has been established and thus avoid the very difficult dilemma of whether or not to terminate a pregnancy or deliver a sick child. Ever since the first application of the technique in 1990 by Verlinsky et al[1] in Chicago with polar body biopsy and in London by Handyside et al that same year[2] with blastomere biopsy, it has been widely applied and the number of indications for PGD has risen rapidly [3, 4]. However, a lower pregnancy rate has been reported in PGD cycles owing to the decreased number of embryos available for transfer[5], which is determined by the inheritance pattern of genetic diseases[6], ovarian response to controlled ovarian stimulation protocols[7–10], embryo micromanipulations performed[11] and so on. As for inherited monogenic diseases, twenty five percent of the embryos are expected to be affected with recessive single-gene disorders, 50% with dominant mutations. Thus, couples need to be aware of the risk of embryo transfer cancellation due to unavailability of transferable embryos after biopsy. With increasing numbers of PGD performed, the need for accurate outcome analysis is required, and there are still questions to be answered regarding its clinical practice.

The aim of our study was to explore factors contribute to the success of PGD cycles for monogenic diseases. The information provided here will assist reproductive specialists, clinicians and genetic counselors to better counsel and treat couples who wish to conceive a healthy child through Intracytoplasmic sperm injection (ICSI) with PGD, and optimize ovarian stimulation and PGD success.

## Materials and Methods

### Patient population

During a 3-year period (January 2009 to December 2012), 184 consecutive ICSI-PGD cycles for monogenic diseases reaching the ovum pick-up and fresh embryo-transfer stage were performed at the Reproductive Medicine Center of The First Affiliated Hospital Of Sun Yat-sen University. The monogenic diseases involved in clinical applications include α-thalassemia, β-thalassemia, spinal muscular atrophy (SMA), Duchennne muscular dystrophy (DMD) and X-linked chronic granulomatous disease (X-CGD). All patients provided written, informed consent of PGD treatment. The elimination of bias in this selection, for the purposes of the present study, was achieved by excluding cycles with cancellation of fresh embryo-transfer, like high risk of ovarian hyperstimulation syndrome (OHSS): women who presented an serum estradiol (E_2_) level exceed 5000 pg/ml on the day of human chorionic gonadotropin (HCG) administration and/or women in whom 25 oocytes or more were retrieved. Other exclusion criteria were endometrium polyp and premature progesterone elevation.

### Stimulation protocols and oocyte retrieval

All patients underwent gonadotropin releasing hormone (GnRH) agonist or antagonist controlled ovarian hyperstimulation (COH) protocols in assisted reproductive technology (ART) cycles. The COH agonist protocol was use of other than a midluteal long GnRH-agonist (Daphiline Beaufour IPSEN, France, 0.8–1.0mg, subcutaneously) suppressive protocol or short GnRH-agonist (Daphiline Beaufour IPSEN, France, 0.05–0.1mg, subcutaneously) suppressive protocol (agonist group). The flexible multidose GnRH-antagonist (cetrorelix, 0.25mg daily, subcutaneously; Serono Laboratories, Aubonne, Switzerland) protocol (antagonist group) was also used. In either protocol, gonadotropins (Gonal-F, Laboratories Serono SA, Switzerland) were administered in variable doses, depending on patient age and/or ovarian responsiveness in previous cycles, and further adjusted according to serum E_2_ levels and vaginal ultrasound measurement of follicular diameter, obtained every two or three days. HCG was administered for final maturation of oocytes when at least three mature(≥17 mm) follicles were identified by transvaginal scan, combined with appropriate serum E_2_ levels. Oocytes were aspirated by the transvaginal ultrasonographic route approximately 36 hours after HCG injection. For luteal phase support, patients received either 40–60mg progesterone intramuscularly (Gestone; Ferring) daily or 600 mg micronized progesterone soft gel vaginal capsules (Utrogestan; Israel) in three divided doses daily, or 90mg progesterone prolonged releasing vaginal gel (Crinone; Merck Serono) daily.

### ICSI, embryo culture and biopsy

ICSI was then performed on metaphase II oocytes as appropriate[12]. After ICSI, oocytes were cultured in G1 medium (Vitrolife, Sweden) for 3 days. In PGD using blastomere biopsy, embryos with at least five blastomeres were biopsied in Ca^2+^/Mg^2+^ free medium under oil (SAGE, BioPharma, US) on the morning of day 3 after oocyte retrieval. Partial zona dissection (PZD) was performed to make a split in the zona pellucida. A sampling micropipette was pushed through the split into the zona to withdraw single cells. Two-cell removal was performed only when the first blastomere was lysed or without a nucleus after biopsy. After biopsy, embryos were rinsed carefully and cultured in blastocyst medium (G2 medium, Vitrolife, Sweden) until transfer[13].

### Genetic diagnosis

#### Fluorescent gap PCR analysis for α-thalassemia Southeast Asia deletion

The biopsied blastomeres were washed three times in sterile phosphate buffer saline (PBS) and transferred into a 0.2 mL tube containing a lysis solution of 0.5 μL 10XPCR Buffer, 0.5 μL 1% Tween-20, 0.5 μL 1% Triton-100, 3.5 μL H2O, and 0.05 μL proteinase K (20 mg/mL) as described previously[13]. For each biopsied blastomere, a blank control was prepared from the final PBS cell wash drop. The positive controls were prepared with 10 pg of purified DNA from the patients. The amplification involved use of three α-thalassemia SEA (Southeast Asia deletion) primers. The S1 and S3 primers flank the SEA deletion, while the S2 primer anneals within the deleted area[14]. The primer sequences and products sizes were shown in Table 1. The Polymerase chain reaction (PCR) procedures were performed as previously described[15]. PCR products were then analyzed on an ABI 3100 Advant genetic analyzer.

**Table 1:** Primer sequences and products sizes for detecting α- and β-thalassemia.

Primers	Primer sequence (5’-3’)	Product size (bp)
S1	GTGTTCTCAGTATTGGAGGGAA	
S2	FAM-GACACGCTTCCAATACGCTTA	Normal:S1 + S2:282
S3	HEX-CTACTGCAGCCTTGAACTCC	Abnormal:S1 + S3:178
A	GGCCAATCTACTCCCAGGAG	
B	ACATCAAGGGTCCCATAGAC	A + B:597
C	ATAACAGTGAATTTCTGG	
D	AAAGCGAACTTAGTGATAC	C + D:362

#### PCR-reverse dot blot (RDB) hybridization analysis for β-thalassemia

We used primers A and B, C and D to detect 16 β-thalassemia mutations (CD41-42, IVS-2nt 654, CD17, −28, CD71/72, −29,βE, CD43, −32, −30, Int, CD14/15, CD27/28, CD1/1,CD1/5, and CD31). The primer sequences and products sizes were shown in Table 1 as well. All PCR were performed following the same reaction system and PCR conditions described previously for PCR-RDB analysis[16].

#### Mutation gene and linkage analysis in SMA, DMD and X-CGD

The biopsied blastomeres were transferred into 0.2 mL tube containing PBS, and used directly for multiple displacement amplification (MDA) (REPLI-g Midi kit, Qiagen, Germany). For monogenic diseases, we conduct haplotype analysis in combination with detecting pathogenic genes directly. The short tandem repeats (STRs) loci located within or on both bounding sides of the pathogenic genes. The number of STRs for each disease and mutation testing methods were listed in Table 2. The information of STR for SMA, DMD and X-CGD were listed in Table 3, Table 4 and Table 5.

**Table 2:** The number of STR for each disease and mutation testing methods.

Disease	Position of causal gene	Number of STR	Methods of mutation testing
SMA	5q13	11	Fluorescent PCR of SMN1 exon7
DMD	Xp21	11	Fluorescent PCR of different exons of DMD gene
X-CGD	Xp21	16	1.Fluorescent PCR:CYBB exon 8
			2.Sequencing

SMA: spinal muscular atrophy; DMD: Duchennne muscular dystrophy; X-CGD: X-linked chronic granulomatous disease.

**Table 3:** The information of STR for SMA.

STR locus	Heterozygosity	Forward primer sequence (5’-3’)	Reverse primer sequence (5’-3’)	Size range (bp)	Fluorescent label
D5S435	0.68	TGGTCCTAAGATAGGGTTGAT	CAAGAGCACAGTTTGGAGTGAG	150	FAM
D5S629	0.82	ACTCGGGAGGCTGAGA	CCGGTTTGTTCCTGTGA	240	HEX
D5S1417	0.79	TTTCAACCCTGAGACATTCAACT	GTGTTAGAATCACCTGGGAAGTG	150	TAMRA
D5S1556	0.98	ATTTACTTTTCCAAGGGGGAGG	CATGTTGCTTAGGCCTCGTCT	105	HEX
D5S610	0.86	GGCAGTGTCCTAAAATCTTTTG	CCTAAACTGAACTTTCAAAGCTG	130	FAM
D5S637	0.72	TGAATCTCAGGGAGTTGTGAA	CTGCATTTAATACTGCAATGAA	245	TAMRA
D5S557	0.46	AAGTGAAACACAGAGGTTGAC	GGTGAATGTTTGATGACCCTA	165	FAM
D5S351	0.74	AAGACCAGTCTATGGCAACAC	GTGAGACCGAAAATGCTGATG	215	HEX
D5S681	0.68	ATCTCTGAGGCTGCACAT	GTCTTTGATGAGATACCG	145	FAM
5’-MAP1B	0.76	TCCTTCTTCCAAAACCAGGGTGAAGCCTC	AAATTTCTAGGATGCTTGCGGGATCTCTTC	135	FAM
D5S641	0.76	AGTTGTGTATTGGAGAATGTTATCA	AGGGACAGTCCACTTCCAGT	265	FAM

**Table 4:** The information of STR for DMD.

STR locus	Heterozygosity	Forward primer sequence (5’-3’)	Reverse primer sequence (5’-3’)	Size range (bp)	Fluorescent label
5’DYSII	0.78	TAGCTAAAAATGTATGAGTA	AATAGTGTTTTCCTAAGGG	90	FAM
STRMP[32]		ACTCACAAGGTTCCAGATATCTAC	CTATCTTTTTGCATTCCAGTTGAG	335	FAM
STR2[32]		CAGCCAGAATATTGACATTACTAC	TCTAGATCCAACTGAAGAGCCTAC	210	FAM
STR4[32]		GCTTTGTCAGAACTTTGTCACCTG	AACTAAGAGTTACATTCCTGTAAG	115	FAM
STR44	0.87	TCCAACATTGGAAATCACATTTCAA	TCATCACAAATAGATGTTTCACAG	180	FAM
STR45	0.88	GAGGCTATAATTCTTTAACTTTGGC	CTCTTTCCCTCTTTATTCATGTTAC	175	HEX
STR49	0.93	CGTTTACCAGCTCAAAATCTCAAC	CATATGATACGATTCGTGTTTTGC	240	HEX
STR50	0.71	AAGGTTCCTCCAGTAACAGATTTGG	TATGCTACATAGTATGTCCTCAGAC	240	FAM
STR62[32]		CACCTATGTTCGCCATCTAGGACA	CAACCATAGTGTTATAAGGCAGAG	399	FAM
STR79GT2 [32]		CCATAGCTTTAGATGTTGTCTGTG	GTTTGAGCAGCCTAGCAGATGTCC	240	FAM
3’CA	0.46	GAAAGATTGTAAACTAAAGTGTGC	GGATGCAAAACAATGCGCTGCCTC	130	FAM

**Table 5:** The information of STR for X-CGD.

STR locus	Heterozygosity	Forward primer sequence (5’-3’)	Reverse primer sequence (5’-3’)	Size range (bp)	Fluorescent label
DXS1214	0.88	TAGAACCCAAATGACAACCA	AAGATAGCAGGCAACAATAAGA	208	FAM
DXS8089	0.90	GGGTGAAATTCCATCACAAA	ACAAATGCAGATGTACAAAAAATA	170	FAM
DXS8113	0.80	CCTCTACATAGGCACATGC	CCAAAGGAGTTATTTGTCACCT	230	FAM
DXS1069	0.82	AGCCTAACCCACATAACAGC	AGCTACTATATTNACCTTGGTCTTG	250	FAM
DXS8085	0.78	TCAAAGAGGTTTTGCCAC	AGATAAAGACATCCTGCCTAGTTC	155	FAM
DXS1055	0.82	ATGGGATACACTGTTCTGGG	TTAAACAATGCACAACTGGG	90	FAM
DXS2507	0.69	GAATCGCTTGAACCCAGAG	AAGGTGACATGAGACTGTGTG	255	HEX
DXS8032	0.83	CATTTTATTTTGCTTTGTATTTGGC	CTCCTAGAACAGTACCTGACACG	180	HEX
DXS1207	0.69	TATTTGCACTTTAACCCTTGTC	CTTCTTTATATCTTATGGGACCACT	170	TAMRA
DXS1216	0.81	TGCCAACAGTGCTAAGGAT	GGGTAGAAGACTTNCCCA	245	HEX
DXS8014	0.80	GGCAAAGTTGTCAGAGGC	CAAATGGCTTGTTCCAGTT	270	FAM
DXS8111	0.81	GTAGGAACAATAAGTTATGCCTTGC	AGATTAAAGCCCTGGCG	165	FAM
DXS8010	0.81	CTGGCCCAAAGGTTAATTTT	AAGGTGGAAGGTCCACTG	160	TAMRA
DXS8060	0.88	CACAGCCATGTCCTAGCATA	ACCAAACTTGTTAGTGACCTGA	135	FAM
DXS1221	0.76	CTTGTGAATTTATTTCAGTTATTG	CCTTAGAAGTGGCCCAG	155	HEX
DXS1226	0.91	CTAAACCCATCTGNCCTC	TTTCCAGCAACTACCTTTCAT	210	HEX

### Embryo transfer procedure

Following genetic diagnosis, one to two unaffected embryos were selected on morphological criteria and transferred into the uterus on the morning of day 4. Supernumerary unaffected embryos were cryopreserved subject to consent by the couple.

### Outcome parameters

Data on patient age, and infertility-treatment-related variables were collected from the database. Ovarian stimulation characteristics, number of oocytes retrieved and number of embryos transferred per cycle were recorded. Fertilization rate, implantation rate and clinical pregnancy rate were calculated.

Fertilization rate was defined as the percentage of fertilized embryos (2PN) in all the mature oocytes. The implantation rate was the proportion of embryo transferred resulting in an intrauterine gestational sac. Pregnancies were confirmed 3 or 4 weeks after embryo transfer (ET), when the serum HCG was elevated. A clinical pregnancy is defined by the presence of one or more gestation sacs in the uterus at transvaginal ultrasound scan. If and when the pregnancy proved to be ongoing, and without any exception, the couple was advised to undergo prenatal diagnosis in order to confirm the preimplantation diagnosis.

### Statistical analysis

Analysis of the data was done using SPSS 13 package programme. Normality of data was tested and statistics was given as median and range. Statistical comparison was carried out by Kolmogorov-Smirnov test. We made use of a linear regression model (Y = B_0_+B_1_X_1_+B_2_X_2_) to relate different explanatory variables (or prognostic factors), such as age, BMI, number of oocytes retrieved, number of biopsied embryos to number of genetically available transferable embryos after PGD. We made use of a binary logistic regression model (logit(P) = B_0_+B_1_X_1_+B_2_X_2_) to relate different explanatory variables including age, BMI, number of genetically available transferable embryos after PGD, number of oocytes retrieved to the outcome of PGD (pregnant or not). A *P* value of less than 0.05 was considered statistically significant.

## Results

184 consecutive ICSI-PGD cycles for monogenic diseases reaching the ovum pick-up and fresh embryo-transfer stage were evaluated. Clinical characteristics of the PGD cycles are shown in Table 6.

**Table 6:** Clinical characteristics of PGD cycles of monogenic diseases.

	Median	Range
Female age(year)	32.0	23.0–41.0
BMI(kg/m^2^)	20.7	16.4–28.7
Basal FSH(IU/L)	5.6	3.2–19.9
Basal LH(IU/L)	3.0	1.0–11.2
Total Gn (IU)	2418.5	900–5250
HCG day E_2_ (ng/ml)	2792.0	103.0–5000.0
No. of Oocytes	14	3–27
No. of MII oocytes	11.5	3–25
NBE	8	3–20
NAE	7	2–20
NDE	7	2–20
NUE	3	1–13
NTE	2	1–3
NFE	0	0–10
NGE	3	1–12

NBE: Number of Biopsied embryos; NAE: Number of Analysed embryos; NDE: Number of Diagnosed embryos; NUE: Number of Unaffected embryos; NTE: Number of Transfered embryos; NFE: Number of Frozen embryos; NGE: Number of Good quality embryos; NS; not statistically significant.

The median dose of gonadotropin required for stimulation was 2418.5 IU (range 900–5250 IU). The median number of cumulus-oocyte complexes (COCs) retrieved was 14 (range 3–27). ICSI was performed on 2206 metaphase II oocytes, and normal fertilization and cleavage rates were 83.4% (1840/2206) and 96.2% (1770/1840), respectively. In the present study, 60.5% (181/299) of day 3 good-quality embryos developed into good-quality embryos on day 4 after biopsy, similarly to the earlier report[17], demonstrating that our biopsy system has no obvious detrimental effect on compaction. A total of 1620 embryos were biopsied, of which 227 were undiagnosed with a diagnostic rate of 86.0%. A total of 740 embryos genetically suitable for transfer (45.7%, 740/1620) were obtained in our retrospective study, similarly to our earlier research[18]. Collectively, 42.9% clinical pregnancy rate (79/184) and 28.5% implantation rate (111/389) were presented.

A linear regression model was used to relate different explanatory variables, such as age, BMI, number of oocytes retrieved, number of biopsied embryos to number of genetically available transferable embryos after PGD, and we found that in the adjusted model, the only two significant factors affecting the number of genetically unaffected embryos were the number of biopsied embryos (coefficient: 0.390, 95%CI 0.317–0.463, *P* = 0.000) and basal FSH level (coefficient: 0.198, 95%CI 0.031–0.365, *P* = 0.021). According to mathematical calculations, this significant impact exists only if basal FSH level smaller than 8.0mmol/L, which represents poor ovarian reservation otherwise. According to the linear regression model, there should be at least four biopsied embryos to obtain at least one unaffected embryos in a PGD system for patients with single gene disorder and under the condition of basal FSH level smaller than 8.0mmol/L.

Comparison between pregnant (N = 79) and non-pregnant subgroup (N = 105) of monogenic disease of PGD cycles were illustrated in Table 7.

**Table 7:** Comparison between pregnant and non-pregnant group of PGD cycles of monogenic diseases.

	Non-pregnant N = 105	Pregnant N = 79	*P* value
Female age(year)	32.0(23.0–41.0)	31.0(23.0–40.0)	NS
BMI(kg/m^2^)	20.5(17.3–28.7)	20.9(16.4–27.0)	NS
Basal FSH(IU/L)	5.6(3.5–9.4)	5.7(3.2–19.9)	NS
Basal LH(IU/L)	3.0(1.3–7.9)	3.0(1.0–11.2)	NS
Total Gn (IU)	2475.0 (1050.0–5250.0)	2250.0 (900.0–4903.0)	NS
HCG day E2(ng/ml)	2706.0(103.0–5000.0)	3128.0(241.0–5000.0)	NS
No. of Oocytes	14(3–27)	13(4–26)	NS
No. of MII oocytes	12(3–25)	11(4–24)	NS
NBE	8(3–20)	9(4–18)	NS
NAE	7(2–20)	8(3–16)	NS
NDE	7(2–20)	8(3–16)	NS
NUE	3(1–10)	4(1–13)	0.03
NTE	2(1–3)	2(1–3)	NS
NFE	0(0–6)	0(0–10)	NS
NGE	2(1–8)	3(1–12)	NS

NBE: Number of Biopsied embryos; NAE: Number of Analysed embryos; NDE: Number of Diagnosed embryos; NUE: Number of Unaffected embryos; NTE: Number of Transfered embryos; NFE: Number of Frozen embryos; NGE: Number of Good quality embryos; NS; not statistically significant.

The number of unaffected embryos of pregnant subgroup was significantly more than that of non-pregnant subgroup (*P* = 0.03).

We made use of a binary logistic regression model to relate different explanatory variables including age, BMI, number of genetically available transferable embryos after PGD, number of oocytes retrieved to the outcome of PGD (pregnant or not). We found that in the adjusted model, the only two significant (*P* < 0.05) factors affecting pregnancy outcome were the number of genetically available transferable embryos after PGD (adjusted OR 1.345, 95% CI 1.148–1.575, *P* = 0.000) and number of oocyte retrieved (adjusted OR 0.934, 95% CI 0.877–0.994, *P* = 0.031). The number of unaffected embryos and oocytes contribute significantly to the outcome of PGD (pregnant or not) in this study.

## Discussion

Preimplantation genetic diagnosis is a novel technique developed to prevent the transmission of inherited diseases to the offspring. Since only genetically unaffected embryos are transferred, the emotional and psychosocial discomfort associated with induced abortion or termination of pregnancy with an affected fetus is overcome. When PGD is added to In vitro fertilization (IVF), additional factors may affect treatment outcome. PGD decreases the numbers of embryos available for transfer by 25 to 81% due to different inheritance pattern[6]. Moreover, the genetic status of the woman may affect her response to ovarian stimulation[9, 19]. As a result, a lower pregnancy rate has been reported in PGD cycles. How to optimize PGD success and ovarian stimulation are key points for clinicians. Couples who come for IVF because of PGD should be routinely investigated for possible infertility factors independent of their genetic diagnosis. Evaluation of the ovarian reservation should be performed by using age, antral follicle count, AMH level and basal FSH levels to allow individual tailoring of the ovarian stimulation protocol to produce a certain amount of unaffected embryos and avoid ovarian hyperstimulation syndrome risks and impaired endometrium receptivity.

Allele drop-out (ADO) is defined as one of two alleles at a heterozygous locus failing to amplify and is one of the main parameters for the evaluation of feasibility of a PGD strategy. ADO is considered as the main cause of misdiagnosis in PGD. In the case of heterozygote embryos, ADO for the normal allele may lead to misdiagnosis as the homozygous mutant embryo; conversely, ADO for the mutant allele may cause misdiagnosis as the normal homozygous embryo. In the current study, ADO rate was estimated from the re-analysis of blastomeres from discarded heterozygous embryos[15] or lymphocytes isolated from peripheral blood of heterozygous carriers[13]. The ADO rates of PGD for monogenic diseases have been calculated in preclinical work-up and clinical PGD cycles in our center and the ADO rates remained at a low level in our clinical practice reported by our group [13, 15, 16, 20, 21]. During a 3-year period (January 2009 to December 2012), we analyzed and detected mutations immediately after single blastomere was lysed without MDA for preimplantation genetic diagnosis of α- and β-thalassemia in our Reproductive Medicine Center. As a result, polymorphic STRs linkage analysis couldn’t be performed due to limited hereditary material. As for SMA, DMD and X-CGD, we detected mutation gene in combination with conducting linkage analysis with polymorphic STR markers which can lower the diagnostic deficiencies due to high level of ADO rates reported by Repli-g approach in case of MDA application and thus, reduce the possibility of choosing ADO-caused abnormal embryos for implantation into the uterus.

Nevertheless, as for autosomal recessive disease, like α-thalassemia, such diagnostic deficiencies will lead to a lower ratio of heterozygote embryos and a reduced number of embryos available for transfer, but homozygous mutant embryos will not be transferred into uterus and lead to any adverse consequences, like Hb Bart’s hydrops fetalis. But affected embryos maybe transferred into uterus due to ADO-caused misdiagnosis as for β-thalassemia and result in serious adverse clinical outcomes. Consequently, we prefer to transfer normal homozygous embryos prior to heterozygote embryos, all of which are suitable for transfer according to morphological criteria after genetic analysis, to lower the possibility of misdiagnosis due to ADO without linkage analysis. As a result we analyze closely linked STR locus in combination with detecting pathogenic genes directly after MDA in PGD cycles for β-thalassemia to avoid misdiagnosis in our clinics ever since 2014. A combination of pathogenic genes detection and linkage analysis for preimplantation genetic diagnosis of monogenic diseases in future studies will reduce the risk of misdiagnosis and identify more genetically unaffected embryos for clinicians as well. With the development of next-generation sequencing technology[22, 23] and microarray technology[24, 25], they will also be considered to improve the diagnostic accuracy and outcome of preimplantation genetic diagnosis for monogenic diseases.

ART success, even without PGD, was demonstrated to be strongly dependent on women’s age and oocyte retrieved. Sunkara et al[26] found a strong association between the number of oocyte retrieved and live birth rate adjusted for age. The number of oocytes that maximized the live birth rate was 15. Live birth rate rose with an increase in numbers of oocytes retrieved up to 15, plateaued between 15 and 20 oocytes, and steadily declined beyond 20 oocytes. Together with other similar findings, it was suggested that the optimal number of oocytes to succeed in ART is ~10 to 15. Considering the decreased proportion of unaffected embryos available for transfer due to inherited diseases, we may ask which explanatory variables contribute significantly to the outcome of PGD (pregnant or not). In our retrospective study, we made use of a binary logistic regression model to relate different explanatory factors to the outcome of PGD to identify significant factors affecting the reproductive outcome of PGD cycles instead of choosing an explanatory factor and determining a cutoff value subjectively. Thus, it is more convincible and eloquent instead of making comparisons between groups simply. Our study showed that the number of unaffected embryos and oocytes contribute significantly to the reproductive outcome of PGD. The number of oocytes significantly contributes to the reproductive outcome of PGD cycles with an OR of 0.934(*P* = 0.031). The pregnancy probability had an approximately 1.6-fold increase in the group with more unaffected embryos compared with the group with fewer unaffected embryos (*P* = 0.000).

The PGD group is burdened with high emotional stress, intense workload and PGD itself is associated with additional high costs. Therefore, it is of great importance to predict the outcome of PGD before it is performed. For years, clinicians are wondering the strategy for poor responders during PGD treatment and whether there is a threshold of a minimum number of oocytes or embryos with which to start PGD or to cancel the cycle. In 1998 Vandervorst et al[27] suggested that it is justifiable to cancel PGD cycles in which it is expected that less than 6 oocytes will be retrieved and that the couple should be informed about the poor prognosis if less than 9 oocytes are retrieved. However, new data challenge the cutoff practice. To better counsel patients on PGD outcome, Tur-Kaspa et al[28] analyzed 560 consecutive IVF-PGD cycles performed at IHR/RGI in Chicago during a 4-year period, they found a low number of oocytes (fewer than seven) was still associated with a fair chance for ET and pregnancy, especially in young patients (<35 years of age). Verpoest et al[29], from the same center as Vandervorst, reevaluated the 1998 findings and analyzed the cumulative reproductive outcome of 1498 couples undergoing PGD in 2009. They found that the number of oocytes at retrieval contributes significantly to the reproductive outcome. However, a critical threshold level cannot be determined. These results suggest that once patients were adequately stimulated and wish to conceive with her own eggs, oocyte retrieval and PGD may be continued even with very low number of oocytes or embryos. Routine canceling of PGD cycles because of low number of oocytes should be reconsidered.

Our study showed that there should be at least four biopsied embryos to obtain at least one unaffected embryos in a PGD system for patients with single gene disorder and under the condition of basal FSH level smaller than 8.0mmol/L. According to mathematical calculations, this significant impact exists only if basal FSH level smaller than 8.0mmol/L, which represents poor ovarian reservation otherwise. These poor ovarian reservation patients should receive individual controlled ovarian hyperstimulation protocol to improve reproductive outcome, taking into account patients' safety and estimated ovarian reserve. These results make sense and assist clinicians to conduct different intensity of COH protocols for patients with different ovarian reservation in clinical practice. In our study, there was only 5 cycles with basal FSH level exceed 8.0mmol/L and further study is necessary to investigate the prognostic factors of pregnancy outcome of PGD cycles with basal FSH level exceed 8.0mmol/L.

Similarly Liu et al[18] demonstrated that pregnancy outcome of PGD cycles by blastomere biopsy with ≥4 good-quality embryos on day 3 are better than that with less than 4 good-quality embryos on day 3. In previous studies, including Liu et al, scholars simply divided cycles into two groups according to a certain factor of clinical importance, like good quality embryos on day 3, and comparisons were made between the two groups. Besides, the cut-off value of the selected factor was determined subjectively. We explore factors contribute to the success of PGD cycles for monogenic diseases from a different point of view through scientific statistical analysis and identify a practical and objective factor to assist reproductive clinicians in controlling the intensity of controlled ovarian hyperstimulation. Obviously, patients with single gene diseases have a relatively higher possibility of obtaining unaffected embryos. According to Genetics, we know that more biopsied embryos, more probability of unaffected embryos for transplantation and higher risks of OHSS. Therefore, individually adjusted ovarian stimulation protocol based on estimated ovarian reserve to achieve maximally effective ovarian stimulation to get at least four biopsied embryos to obtain at least one unaffected embryos for transfer in a PGD system instead of getting as many oocytes and biopsied embryos as possible, is a foremost concern in PGD cycles. The number of biopsied embryos is a practical and common factor in clinical practice and the result will assist reproductive clinicians to better treat couples and control the intensity of COH protocols to avoid iatrogenic complications of assisted reproductive treatment, like OHSS and multiple pregnancies. Moreover, if only a low number (< 4) of biopsied embryos are available on day 3, patients may have no unaffected embryos for transfer and have poor outcomes. There may be several other options available for these patients instead of canceling this treatment cycle, such as pooling the good-quality embryos by cryopreservation before biopsy for the next PGD cycle, direct embryo transfer followed by prenatal diagnosis, for the purpose of decreasing the cost of biopsy and diagnosis and increasing the chances of embryo transfer.

The limitations of this study include its retrospective nature and the small number of patients in PGD cycles. What’s more, we performed embryo biopsy at the cleavage stage, followed by fresh embryo transfer, which is the most common approach for PGD of monogenic diseases[30]. Nowadays, clinicians prefer blastocyst biopsy, vitrification and thawed embryo transfer for PGD of monogenic diseases[31], which permits sufficient time for transportation of specimens and molecular diagnosis. And we are also observing the effectiveness of this strategy.

## Conclusion

Our results provide a basis for correct counseling of couples undergoing ICSI and PGD, and may serve as a guide in their decision whether or not to undergo this treatment. In summary, there should be at least four biopsied embryos to obtain at least one unaffected embryos in a PGD system for patients with single gene disorder and under the condition of basal FSH level smaller than 8.0mmol/L. Moreover, if only a low number (< 4) of biopsied embryos are available on day 3, patients may have no unaffected embryos for transfer and have poor outcomes. There may be several other options available for these patients instead of canceling this treatment cycle.
